# Case report: Effective treatment of posttraumatic aggression in a child affected by TBI treated with long-acting injectable paliperidone: a case study and literature review

**DOI:** 10.3389/fpsyt.2026.1772148

**Published:** 2026-04-29

**Authors:** Abdullah Al Ghailani, Samir Al Adawi, Hassan Mirza

**Affiliations:** 1 Oman Medical Specialty Board, Psychiatry, Muscat, Oman; 2 Department of Behavioral Medicine, Sultan Qaboos University Hospital, College of Medicine & Health Sciences, Sultan Qaboos University, Muscat, Oman

**Keywords:** long-acting injectable antipsychotics, Oman, paliperidone palmitate, pediatric neuropsychiatry, post-traumatic confusional state, traumatic brain injury

## Abstract

Post-traumatic confusional state (PTCS) is a disabling sequel of pediatric traumatic brain injury (TBI), with little guidance on pharmacologic management when aggression and poor impulse control dominate and adherence is poor. We describe a 9-year-old boy with severe TBI who evolved from coma to a prolonged PTCS characterized by agitation, externalizing behaviors, and dysexecutive symptoms. Multiple oral agents—risperidone, methylphenidate, olanzapine, and aripiprazole—were trialed over several years with minimal benefit and poor adherence. Following an acute agitation episode in 2022, long-acting injectable (LAI) paliperidone palmitate was initiated (100 mg then 150 mg monthly) with adjunct nightly risperidone 2 mg. Over two years, aggression markedly decreased and functional status improved from Rancho Los Amigos Level IV (“confused–agitated”) to Level VIII (“purposeful–appropriate”). Cognitive testing at age 19 showed average intellectual ability but persistent executive dysfunction. Adverse effects were limited to weight gain and asymptomatic hyperprolactinemia; no extrapyramidal or cardiac events occurred. While spontaneous recovery and developmental maturation cannot be excluded, this case suggests LAI paliperidone can be a practical option to stabilize severe PTCS-related behavioral dysregulation when oral adherence fails. Larger, controlled studies are needed to clarify efficacy, safety, and long-term neurocognitive impact of LAI antipsychotics in pediatric TBI.

## Introduction

Traumatic brain injury (TBI) is a major global health problem that affects millions of people each year and often leads to complex cognitive and psychological complications. Concussion is associated with various neuropsychiatric and neurodegenerative disorders (1). TBI cases range in severity from mild to severe, with moderate to severe injuries frequently associated with long-term challenges such as physical, emotional, and cognitive functioning. The cascade of neurometabolic pathological changes tends to occur during acute and chronic TBI. According to the existing literature, both acute and chronic TBI are characterized by dysregulation of excitatory amino acids, neuroinflammation, oxidative stress, and imbalances in neurotransmitter systems (2). The understanding of neurometabolic changes and the resultant neurotransmitter imbalance has led to a surge of interest in the use of some existing medications to help survivors of TBI regain some of the lost function. To date, although not exclusive, there is growing interest in compounds targeting dysregulation in glutamatergic, GABAergic, and monoaminergic networks (3). In support of this view, several studies have shown that certain pharmacological agents can ameliorate some post-TBI symptoms. The primary focus has been on the use of pharmacological agents that have been hypothesized to act as agonists for glutamatergic, GABAergic, and monoaminergic dysfunction (4). These agonists have been lauded for kick-starting and, to some extent, reversing the post-traumatic phenotype, such as those with psychomotor retardation, negativistic behavior, and apathetic-abulia characteristics (5). However, when it comes to people with TBI marked with behavioral dysregulation, impaired impulse control, agitation, and externalization behavioral problems, various compounds have been used. There is always the challenge of whether pharmacological agents that work as antagonists have the potential to impede what often tends to be ‘spontaneous recovery’. In addition to offending social modesty and burdening the family, behavioral dysregulation can prolong hospital stays, delay rehabilitation progress, and ultimately limit the ability of patients to regain functional independence (6, 7).

At this moment, we present a case report from Oman using paliperidone, an atypical antipsychotic that resulted in a significant reduction in post-traumatic confusional state. Oman, a country in the Arabian Gulf, is not immune to the vagaries of TBI. The population of this area comprises numerous young people who are often prone to TBI (8, 9). The mechanism of action includes modulation of the dopamine D2 receptor to block ‘overactive dopamine pathways’, antagonism of the serotonin 5-HT2A receptor, and weak antagonism at α1-adrenergic receptors and histamine H1 receptors (10). In this case report, long-acting injectable paliperidone palmitate was used. Only a few reports exist on the use of paliperidone palmitate in children and adolescent survivors of TBI, with some exceptions (11).

## Literature review: medical management of post-TBI aggression

A targeted search was conducted in major biomedical databases to identify relevant literature on the pharmacological management of agitation in traumatic brain injury (TBI). The databases searched included PubMed/MEDLINE, Embase, Cochrane Library, PsycINFO, and Scopus. The search strategy used a combination of keywords and Medical Subject Headings (MeSH) terms, including “Traumatic Brain Injury (TBI),” “agitation,” “aggression,” “pharmacological management,” and specific medication classes such as beta-blockers, mood stabilizers, antipsychotics, stimulants, and anticonvulsants. Additional filters were applied to prioritize peer-reviewed studies, clinical trials, and review articles published in English that addressed medication effectiveness, safety, and long-term outcomes in pediatric and adolescent patients, or provided comparisons to adult populations. Articles were included if they discussed pharmacological interventions for post-TBI behavioral disturbances, with an emphasis on age-appropriate considerations.

Effective pharmacological management of posttraumatic aggression requires a clear understanding of available treatment options for agitation and behavioral disturbances following TBI. Various medications, including beta-blockers, mood stabilizers, antiepileptics, and atypical antipsychotics, target different aspects of TBI-related neuropsychiatric dysfunction, as summarized in **Table 1**.

**Table 1:** Summary of pharmacological agents trialed for post-TBI agitation and aggression, with their primary uses, advantages, disadvantages, and key references.

Drug class	Medications	Primary use	Advantages	Limitations and monitoring
Dopaminergic agents	Amantadine* (12–14)	Arousal, cognition, irritability, and aggression	May improve attention and processing speed; inexpensive; oral option	Insomnia, irritability, hallucinations, and lower seizure threshold; monitor mood and seizures
Atypical antipsychotics	Olanzapine*; quetiapine; risperidone; paliperidone; ziprasidone* (15–19)	Acute or severe agitation and aggression	Multiple formulations, including long-acting injectable (LAI) options; broad anti-agitation effect	Sedation, metabolic effects, hyperprolactinemia, corrected QT interval (QTc) prolongation, and possible cognitive dulling; monitor weight, lipids, prolactin, and electrocardiogram (ECG)
Typical antipsychotics	Haloperidol (20, 21)	Rapid control of severe agitation	Fast intramuscular (IM) onset; inexpensive; widely available	Extrapyramidal symptoms (EPS), neuroleptic malignant syndrome (NMS), and QTc prolongation; may hinder neurorecovery; monitor ECG, rigidity, and creatine kinase (CK)
Benzodiazepines	Midazolam; lorazepam; clonazepam; diazepam (22)	Acute agitation or procedural sedation	Very rapid onset; anticonvulsant effect	Paradoxical agitation, delirium, respiratory depression, and dependence; best for short-term use
Antidepressants (selective serotonin reuptake inhibitors [SSRIs]/tricyclic antidepressants [TCAs])	Sertraline; *amitriptyline (23–26)	Emotional dysregulation, irritability, and depression	Useful for comorbid mood and anxiety symptoms; SSRIs generally better tolerated	Delayed onset; activation or mania; serotonin syndrome with SSRIs; anticholinergic and cardiac adverse effects with TCAs; monitor mood and ECG with TCAs
Beta-blockers	Propranolol; pindolol (27–29)	Reduction of aggression through noradrenergic blockade	Some evidence for reducing aggression; lowers heart rate and blood pressure; possible mortality benefit in traumatic brain injury (TBI) cohorts	Hypotension, bradycardia, bronchospasm, and depression; monitor vital signs
Psychostimulants and cognitive enhancers	*Methylphenidate; dextroamphetamine; *donepezil (30–34)	Cognitive deficits and attention problems	May improve attention and processing speed; may support rehabilitation participation	May worsen impulsivity or agitation; increased blood pressure and heart rate, insomnia, and appetite loss; monitor vital signs and behavior
Mood stabilizers	*Oxcarbazepine; sodium valproate (divalproex); lithium (35–37)	Aggression and mood lability	May help when affective instability is prominent; multiple mechanisms	Sedation; hepatotoxicity with sodium valproate; hyponatremia with oxcarbazepine; renal and thyroid adverse effects with lithium; therapeutic drug monitoring (TDM) required where applicable
Hormonal modulators	Alkylphenols; estrogen (38, 39)	Experimental use; modulation of neuroinflammation	Possible anti-inflammatory and neuroprotective effects, with limited data	Thromboembolism, endocrine adverse effects, and mood changes; mainly research setting or last-line use

Agents trialed in pediatric/adolescent populations are marked with *.

Despite extensive research in adult populations, pharmacological outcomes in adolescent patients remain understudied. Most clinical trials and randomized controlled studies focus on adults, with limited large-scale research on safety, efficacy, and long-term effects in younger patients. Given the unique neurodevelopmental factors in children and adolescents, direct extrapolation from adult data may be inappropriate, as differences in drug metabolism, cognitive maturation, and neurological development must be considered (40).

A subset of medications (denoted with * in **Table 1**) has been trialled in pediatric and adolescent populations, though evidence remains limited and primarily case-based. While some agents show promise in managing agitation, impulsivity, and cognitive dysfunction, concerns persist regarding long-term safety, tolerability, and adherence.

## Case

AA, a 9-year-old boy, was hit by a car in 2014 and sustained intracranial bleeding, seizures, and multiple physical and orthopedic complications. The accident left him in a coma for three months and required four months of hospitalization. During hospitalization, he progressed from coma to a vegetative state, also termed unresponsive wakefulness syndrome, and later to a minimally conscious state. Seizures were noted early in the course, and antiepileptic treatment was initiated. After a prolonged critical illness, he underwent rehabilitation but showed poor engagement with prescribed therapies. His behavior remained difficult to manage in structured settings. Following emergence from the minimally conscious state, he entered a prolonged confusional phase marked by inattention, disorientation, agitation, impulsivity, and poor participation in rehabilitation. Although basic function gradually improved, persistent cognitive and behavioral impairment continued to limit rehabilitation and later school reintegration.

Approximately 1 year after the accident, he was referred to the child and adolescent psychiatry clinic for assessment of functional impairment and disability. During the initial consultation, he remained in a confusional state characterized by disorientation, impaired attention, fluctuation in symptoms, and behavioral dysregulation. Using the Rancho Los Amigos Levels of Cognitive Functioning Scale (RLA-LOCF) (41), his global score was below Level IV, indicating a confused and agitated state. He displayed non-purposeful and aggressive behavior and was unable to cooperate with treatment because of cognitive symptoms and behavioral problems. Accordingly, input from a licensed applied behavior analyst was sought to address his confusion, impulsivity, and agitation. However, the clinical team considered that his severe externalizing behavior and limited engagement would substantially limit the effectiveness of applied behavior analysis as a standalone intervention. Pharmacotherapy was therefore considered.

At specialist neuropsychiatric assessment, the presentation was dominated by severe cognitive-behavioral dysregulation rather than focal psychotic symptoms. He was distractible, disoriented, impulsive, poorly redirectable, and at times aggressively disorganized. There was no sustained evidence of a primary mood disorder, formal thought disorder, or persistent hallucinations.

Diagnostic assessment. The diagnosis was based on longitudinal multidisciplinary assessment integrating the history of severe traumatic brain injury, post-injury course, serial behavioral observation, functional impairment across settings, RLA-LOCF staging, neurological review, electroencephalogram (EEG) findings, and later formal neuropsychological testing. The working formulation was prolonged post-traumatic confusional state with severe behavioral dysregulation and residual dysexecutive syndrome after severe pediatric traumatic brain injury. Diagnostic challenges included the long interval since injury, poor adherence, and overlap between injury-related symptoms and developmental change. Differential diagnoses considered were primary psychotic disorder, mood disorder with behavioral dyscontrol, seizure-related behavioral disturbance, and medication-induced symptoms; these were considered less likely based on the longitudinal course, absence of sustained psychotic or mood syndromes, EEG findings, and the overall neurobehavioral profile. Prognosis was guarded because of severe initial injury, prolonged disorder of consciousness, chronic agitation, and persistent executive dysfunction, although later functional gains suggested meaningful recovery despite residual deficits. The chronological progression of the episode of care is summarized in **Table 2**.

**Table 2:** Timeline of the episode of care.

Year/age	Clinical stage	Key findings	Intervention	Outcome
2014/9 years	Severe traumatic brain injury	Intracranial bleeding, seizures, coma → vegetative state/unresponsive wakefulness syndrome → minimally conscious state	Antiepileptic treatment, hospitalization, rehabilitation	Persistent neurobehavioral impairment
2014–2015/9–10 years	Prolonged confusional phase	Inattention, disorientation, agitation, impulsivity, poor rehabilitation engagement	Multidisciplinary follow-up and rehabilitation	Limited functional progress
2015/10 years	Confusional-agitated state	RLA-LOCF below Level IV; poor treatment cooperation	Risperidone (trialed for several weeks), then methylphenidate 10 mg (trialed for a short period)	No meaningful improvement, poor compliance
2015–2019	Persistent behavioral dysregulation	Ongoing aggression and disruptive behavior	Traditional remedies	No sustained benefits
2020/15 years	Persistent PTCS	RLA-LOCF remained at Level IV (confused-agitated)	Olanzapine 5–10 mg (brief trial)	Stopped because of sedation
2020/15 years	Persistent PTCS	Poor adherence; worsening behavior	Aripiprazole 7.5 mg (trialed over a 2–3 weeks)	Discontinued
2020–2021/15–16 years	Home and school dysfunction	Aggression toward peers and family; unsafe wandering; EEG: frontal slowing without epileptiform discharges	Special-needs school placement; follow-up	Dysregulation persisted
March 2022/17 years	Acute behavioral crisis	Severe agitation and aggression	Intramuscular haloperidol; paliperidone palmitate 100 mg then 150 mg monthly; risperidone 2 mg nightly	Early stabilization
2022–2024/17–19 years	Sustained improvement	Fewer aggressive outbursts; improved structured engagement; BMI 20.2→25.5; asymptomatic hyperprolactinemia; no major adverse effects	Continued LAI paliperidone, adjunct risperidone, monitoring	Sustained behavioral stabilization
2024/19 years	Purposeful-appropriate stage	RLA-LOCF Level VIII; average intellectual ability with persistent executive dysfunction and relatively preserved memory (42–47)	Continued follow-up	Improved daily functioning with residual dysexecutive deficits

The initial pharmacotherapy in 2015 consisted of risperidone, started at 0.25 mg twice daily and later increased to three times daily. Despite several weeks of treatment, the family reported no meaningful clinical or functional improvement. Methylphenidate 10 mg was then trialed for a short period, again without notable improvement in externalizing or disruptive behavior. Both medications were therefore discontinued because of insufficient therapeutic benefit. The family subsequently sought traditional healing using herbal and mineral remedies, but the behavioral problems persisted.

When AA returned in 2020, he continued to show post-traumatic confusional state with stereotypical behavior. Repeat assessment using the RLA-LOCF placed him at Level IV, indicating a confused and agitated state. Olanzapine was initiated at 5 mg and titrated to 10 mg, but was stopped after a brief trial because of excessive sedation. Aripiprazole 7.5 mg was then trialed over the following weeks because of its more favorable side-effect profile; however, adherence remained poor, with worsening behavior and repeated refusal to take the medication.

During this period, behavioral dysregulation, impaired impulse control, agitation, and externalizing behaviors continued to escalate. He was reenrolled in a school program for students with special needs, where reports documented aggression toward peers and declining academic performance. At home, aggression toward family members intensified, and he engaged in risky behaviors, including inappropriate wandering and threatening others with sharp objects. Neurological evaluation, including EEG studies, showed occasional frontal slowing without epileptiform discharges, which did not suggest an active epileptiform process as the main driver of the presentation.

In March 2022, AA was brought to the emergency department in an acutely agitated and aggressive state, requiring intramuscular haloperidol for immediate behavioral control. Given the repeated difficulty maintaining oral treatment, his regimen was changed to long-acting injectable paliperidone palmitate. An initial dose of 100 mg was increased to 150 mg monthly to optimize response, with adjunct risperidone 2 mg at night. Following this change, the family reported marked improvement in aggression and overall behavioral stability, although intermittent risky behaviors, such as running outside and verbal aggression, still occurred.

Over the subsequent two years, follow-up documented better impulse control, fewer aggressive outbursts, and improved tolerance of structured interactions. Body mass index increased from 20.2 at treatment initiation to 25.5. Apart from weight gain and asymptomatic hyperprolactinemia, no extrapyramidal symptoms, clinically significant corrected QT interval (QTc) prolongation, treatment-related seizure recurrence, or hematologic adverse events were observed. Monitoring included serial body mass index, fasting lipids and glucose, prolactin, and electrocardiograms (ECGs).

By 2024, repeat RLA-LOCF assessment placed AA at Level VIII, indicating purposeful and appropriate functioning. He was alert and oriented and showed good recall of recent and remote events. The family reported that he had become independent in daily activities at home. A formal psychometric evaluation at the age of 19 years in 2024 indicated that he had average intellectual ability on Raven’s Progressive Matrices (42). However, his performance on executive function measures, including the Tower of London (43), Trail Making Test (44), and Wisconsin Card Sorting Test (45), was suboptimal. In contrast, he performed within the adequate range on memory measures, including the California Verbal Learning Test (46) and Rey-Osterrieth Complex Figure Test (47). Thus, his residual neurocognitive profile was characterized more by dysexecutive dysfunction than by amnesia.

## Discussion

This case describes a male patient who sustained severe traumatic brain injury at age 9 and subsequently developed chronic posttraumatic confusional state (PTCS) with severe aggression and impulsivity. Multiple oral psychotropics were limited by inadequate response, poor tolerability, or difficulty maintaining consistent oral treatment. Monthly long-acting injectable (LAI) paliperidone palmitate, together with low-dose nocturnal risperidone, was associated with sustained behavioral stabilization and functional recovery from RLA-LOCF Level IV to Level VIII over two years, although dysexecutive deficits persisted.

After severe TBI, a common recovery trajectory progresses from coma to vegetative state/nonresponsive wakefulness syndrome, minimally conscious state, and finally recovery to a confusional state marked by disorientation, significant impairments in cognitive function, inattention, and a constellation of clinical symptoms, including behavioral dysregulation and fluctuation in symptoms. In the existing literature, these findings constitute the hallmark of what is known as the posttraumatic confusional state (PTCS) (48). The present case has all the hallmarks of PTCS. These manifestations should also be interpreted within a developmental framework. As the patient progressed through later childhood and adolescence, persistent executive dysfunction and behavioral dysregulation likely reflected a combination of injury-related impairment and ongoing neurodevelopmental maturation.

The case of AA highlights the complex recovery trajectory and challenges after severe TBI and prolonged PTCS. Despite initial difficulties, AA demonstrated notable progress in certain functional areas over time. AA initially exhibited severe cognitive impairment, behavioral dysregulation, and agitation, consistent with a confusional state (RLA-LOCF Level IV). His erratic and aggressive behavior presented significant challenges to therapeutic and educational interventions. Persistent dysexecutive symptoms, such as impaired impulse control and difficulties in planning and problem-solving, remained prominent. However, amnesia was not a defining issue, as memory performance had improved to normal ranges. As often reported in the literature, in the amnesic-executive dichotomy, the present case appears to have overcome the broad amnesia syndrome but remained marked by executive dysfunction (49). Behavioral dysregulation is clinically significant in this context because it directly affects rehabilitation participation, school reintegration, family burden, and exposure to risk.

Regarding pharmacological interventions, multiple pharmacological trials (eg, risperidone, methylphenidate, olanzapine, and aripiprazole) had limited success, because of inadequate response, poor tolerability, or difficulty maintaining consistent oral treatment. However, the transition to monthly long-acting injectable (LAI) paliperidone palmitate, supplemented with nightly risperidone, produced sustained stabilization of PTCS. By 2024, he reached RLA-LOCF Level VIII (“purposeful–appropriate”) with intellectual recovery and independence in daily living, although executive deficits persisted and limited full social/educational reintegration. Treatment response was supported by both the longitudinal clinical course and the available quantitative markers. Functionally, the patient improved from RLA-LOCF below Level IV during the confusional-agitated phase to Level VIII by 2024. This was accompanied by sustained reduction in aggressive outbursts over approximately two years of monthly treatment, together with gains in personal hygiene, social reciprocity, and independence at home.

The choice of paliperidone palmitate was guided by limited benefit and poor tolerability with previous oral agents, together with difficulty maintaining consistent oral treatment. At the time of the acute behavioral presentation in 2022, the main priorities were sustained reduction in aggression and simplification of the regimen. A monthly long-acting injectable formulation was therefore favored. This choice was also informed by our clinical experience, in which paliperidone has shown a good response in our patient population. It was selected not as a first-line treatment for post-TBI agitation, but as a pragmatic option in this refractory case after unsuccessful oral strategies.

Although paliperidone palmitate was the primary intervention, a low nightly dose of risperidone was maintained to smooth breakthrough symptoms and sleep-related agitation. This adjunct complicates strict attribution of improvement to the LAI alone. Pharmacodynamically, both agents share D₂/5-HT₂A antagonism; therefore, a synergistic or simply additive effect is plausible. However, the marked behavioral stabilization was observed after initiation of the LAI, although causality cannot be inferred from a single uncontrolled case. Neurobiological processes such as neurogenesis, synaptogenesis, and experience-dependent plasticity facilitate post-TBI reorganization and cognitive recovery (50). In adolescence, frontostriatal and prefrontal networks continue maturing into the mid-20s, supporting gains in impulse control and planning (51). Distinguishing medication effects from this developmental trajectory is therefore difficult. Notably, the most marked behavioral stabilization in our case occurred between 2022 and 2024 (ages 17–19), a window compatible with both late adolescent cortical refinement and active pharmacotherapy. Future reports should align symptom trajectories with developmental milestones (e.g., serial executive-function testing) to sharpen causal inference. Spontaneous recovery is well documented in individuals with TBI, who often regain lost functions over time (52). Because the injury in this case occurred at the age of 9 years, it is plausible that the subsequent expression of PTCS reflected not only the neurological consequences of severe TBI but also the influence of developmental stage. Pubertal transition may have amplified features such as impulsivity, behavioral dysregulation, and risk-taking (53), whereas increasing maturity may have contributed to later reduction in these behaviors. As reported in the clinical literature, some neural pathways involved in decision-making are not fully mature until the mid-20s (54). This delayed maturation may help explain why typical adolescent behaviors can overlap with, or modulate, the clinical expression of PTCS.

The neuropsychological profile in this case was more consistent with executive dysfunction than with an amnesic syndrome (55, 56). Third, if one lumps the common compound used in TBI for neuropsychiatric sequelae into stimulant versus non-stimulant compounds, though largely case reports, there is **some** evidence that neurostimulants may facilitate recovery although the mechanisms are still speculative (57). However, risperidone and paliperidone, which are neuroleptics, are generally used cautiously in TBI because of concern that they might impede recovery. Our findings enter an ongoing debate; preclinical and some clinical reports caution that dopamine antagonism may blunt neurorehabilitation (58). Conversely, case-based and limited clinical data (11) show pragmatic benefits for severe agitation. This case suggests that, when treatment selection is individualized and monitoring is maintained, LAI antipsychotics may support behavioral stabilization in selected refractory cases. Thus, treatment decisions should be individualized, balancing neuroplasticity concerns against the real-world risks of uncontrolled aggression and inconsistent treatment.

Unlike the previous study that reported exclusive use of paliperidone palmitate, the present case appears to have shown improvement with 2 mg of risperidone at night. The patient showed notable behavioral improvement after paliperidone palmitate administration. Although research on LAI use in adolescents with TBI remains limited, existing studies on LAI agents such as risperidone and paliperidone indicate that these treatments are generally safe and well tolerated (59, 60). To our knowledge, this remains the only reported case describing LAI paliperidone palmitate in prolonged PTCS in a pediatric or adolescent TBI context. Ensuring medication compliance is crucial for managing post-TBI behavioral disturbances, and LAIs offer a practical approach to consistent dosing and sustained symptom control (61). Studies have shown that monthly paliperidone injections can improve medication compliance and behavior outcomes in patients who struggle with medication compliance (62). The safety profile, with a low risk of metabolic and extrapyramidal side effects (63), supports its suitability as a long-term option to manage TBI-related aggression in selected cases under careful monitoring.

At the same time, this case has several limitations. Objective behavioral rating scales were not used, which limits precise quantification of symptom change. The treatment course was not controlled or blinded, and adjunct nightly risperidone complicates strict attribution of benefit to paliperidone palmitate alone. Spontaneous neurological recovery, developmental maturation, rehabilitation exposure, and environmental factors may all have contributed to improvement over time (50–54). Accordingly, causal inference remains limited. A formal patient perspective was not obtained because of the patient’s cognitive and behavioral limitations during much of the course. However, the family caregiver’s perspective remained an important collateral source regarding treatment adherence, perceived benefit, tolerability, and day-to-day functional change.

This case report has been prepared in accordance with the CARE (CAse REport) guidelines to ensure transparency, completeness, and accuracy in reporting. The structured approach follows established standards for case report documentation, providing a clear and comprehensive account of the patient’s clinical course, diagnosis, intervention, and outcomes. Adherence to these guidelines enhances the reproducibility and credibility of the findings, aligning with best practices in medical case reporting (64).

## Conclusion

This case highlights the complexity of managing severe neuropsychiatric sequelae after pediatric traumatic brain injury, particularly when prolonged post-traumatic confusional state is accompanied by aggression, impulsivity, and persistent executive dysfunction. In this refractory case, long-acting injectable paliperidone palmitate was associated with sustained behavioral stabilization, reduction in aggression, and functional improvement over follow-up, after previous oral strategies had been limited by inadequate response, poor tolerability, or difficulty maintaining consistent treatment.

At the same time, interpretation should remain cautious because recovery in prolonged post-traumatic confusional state is multifactorial and may also reflect spontaneous neurological recovery, developmental maturation, rehabilitation exposure, and environmental influences. Accordingly, this case should not be interpreted as supporting routine use of long-acting injectable antipsychotics in post-traumatic agitation, but rather as suggesting that paliperidone palmitate may be a practical option in selected difficult-to-treat cases when carefully monitored and individualized risk-benefit considerations favor this approach.

The clinical value of this report lies in its description of treatment selection, adherence-related decision-making, tolerability, safety monitoring, and longitudinal functional outcome in a pediatric population for whom evidence remains limited. Further controlled studies are needed to clarify efficacy, safety, and long-term neurocognitive impact in children and adolescents with traumatic brain injury.

## Data Availability

The original contributions presented in the study are included in the article/supplementary material. Further inquiries can be directed to the corresponding author.
