# Supplementary material for: Case report: Effective treatment of posttraumatic aggression in a child affected by TBI treated with long-acting injectable paliperidone: a case study and literature review
Source: Front Psychiatry. 2026 Apr 29;17:1772148. doi: 10.3389/fpsyt.2026.1772148 (PMC13168192; doi:10.3389/fpsyt.2026.1772148)
Supplement: Supplementary file 1 [file SupplementaryFile1.docx]

**CARE Checklist:**

| **Item** | **Description** | **Response / location in manuscript** |
| --- | --- | --- |
| **1** | **Title** | Effective treatment of posttraumatic aggression in a child affected by traumatic brain injury treated with long-acting injectable paliperidone: a case report and literature review. |
| **2** | **Key words** | Traumatic brain injury; posttraumatic confusional state; paliperidone palmitate; behavioral dysregulation; case report. |
| **3a** | **Abstract: uniqueness / contribution** | Rare use of long-acting injectable paliperidone palmitate in prolonged pediatric posttraumatic confusional state; highlights practical relevance in a difficult-to-treat case. |
| **3b** | **Abstract: main symptoms / findings** | Severe traumatic brain injury, prolonged posttraumatic confusional state, aggression, impulsivity, cognitive-behavioral dysregulation, poor oral treatment maintenance. |
| **3c** | **Abstract: diagnosis / intervention / outcomes** | Prolonged posttraumatic confusional state; oral trials followed by paliperidone palmitate plus low-dose risperidone; improvement from RLA-LOCF below Level IV to Level VIII. |
| **3d** | **Abstract: conclusion / take-away** | Long-acting injectable paliperidone palmitate may be useful in selected refractory pediatric traumatic brain injury cases with close monitoring. |
| **4** | **Introduction** | Summarizes traumatic brain injury neuropsychiatric sequelae, posttraumatic confusional state, behavioral dysregulation, and the limited evidence for this treatment approach. |
| **5a** | **Patient information** | 9-year-old male, anonymized as AA. |
| **5b** | **Main concerns / symptoms** | Aggression, agitation, impulsivity, severe behavioral dysregulation, poor rehabilitation engagement, difficulty maintaining oral treatment. |
| **5c** | **Medical / family / psychosocial history** | Severe traumatic brain injury after motor vehicle collision; family closely involved in care; major behavioral burden at home and school. |
| **5d** | **Past interventions / outcomes** | Risperidone, methylphenidate, olanzapine, and aripiprazole were trialed with inadequate response, poor tolerability, or poor oral treatment continuity; traditional remedies showed no sustained benefit. |
| **6** | **Clinical findings** | Prolonged confusional phase with inattention, disorientation, agitation, impulsivity, poor treatment cooperation, and severe behavioral dysregulation. |
| **7** | **Timeline** | Summarized in Table 2. |
| **8a** | **Diagnostic methods** | Clinical assessment, RLA-LOCF, electroencephalogram, and formal neuropsychological testing. |
| **8b** | **Diagnostic challenges** | Long interval since injury, developmental overlap, fluctuating symptoms, and difficulty maintaining oral treatment. |
| **8c** | **Diagnostic reasoning / differentials** | Working diagnosis: prolonged posttraumatic confusional state; differentials included primary psychotic disorder, mood disorder with behavioral dyscontrol, seizure-related disturbance, and medication-induced symptoms. |
| **8d** | **Prognosis** | Guarded initially because of severe injury and prolonged disorder of consciousness; later meaningful recovery despite residual dysexecutive deficits - Discussion |
| **9a** | **Therapeutic intervention** | Pharmacologic management with long-acting injectable paliperidone palmitate and adjunct low-dose risperidone after unsuccessful oral trials. |
| **9b** | **Intervention administration** | Paliperidone palmitate 100 mg monthly, increased to 150 mg monthly; risperidone 2 mg nightly. |
| **9c** | **Changes in intervention / rationale** | Oral agents were discontinued because of inadequate response, poor tolerability, or poor treatment continuity; long-acting injectable therapy chosen as a pragmatic option in a refractory case. |
| **10a** | **Follow-up / outcomes** | Reduced aggression, improved impulse control, better structured engagement, improved hygiene, social reciprocity, and independence; RLA-LOCF improved to Level VIII. |
| **10b** | **Follow-up test results** | Average intellectual ability with persistent executive dysfunction and relatively preserved memory on formal testing. |
| **10c** | **Adherence / tolerability** | Improved treatment continuity after monthly injectable treatment; acceptable tolerability with ongoing monitoring. |
| **10d** | **Adverse events** | Weight gain and asymptomatic hyperprolactinemia; no extrapyramidal symptoms, clinically significant corrected QT interval prolongation, hematologic adverse events, or treatment-related seizure recurrence. |
| **11a** | **Discussion: strengths / limitations** | Longitudinal follow-up and practical treatment relevance; limitations include lack of standardized behavioral scales, uncontrolled course, adjunct risperidone, and possible contribution of spontaneous recovery, maturation, rehabilitation, and environment. |
| **11b** | **Discussion: literature** | Case contextualized within literature on posttraumatic confusional state, pediatric traumatic brain injury behavioral dysregulation, developmental recovery, and long-acting injectable antipsychotics. |
| **11c** | **Discussion: rationale / interpretation** | Explains treatment selection, mechanism, safety monitoring, and the need for cautious interpretation because causality cannot be established in a single uncontrolled case. |
| **11d** | **Discussion: takeaway lesson** | Long-acting injectable antipsychotics may be useful in selected refractory cases, but require individualized risk-benefit assessment and monitoring. |
| **12** | **Patient / family perspective** | Formal patient perspective not feasible because of cognitive and behavioral limitations; caregiver perspective used as collateral source regarding adherence, benefit, tolerability, and daily function. |
| **13** | **Informed consent** | Written informed consent obtained from the caregiver; report anonymized. |
